# Systemically-delivered biodegradable PLGA alters gut microbiota and induces transcriptomic reprogramming in the liver in an obesity mouse model

**DOI:** 10.1038/s41598-020-69745-x

**Published:** 2020-08-14

**Authors:** Alice Chaplin, Huiyun Gao, Courteney Asase, Palanivel Rengasamy, Bongsoo Park, Danielle Skander, Gürkan Bebek, Sanjay Rajagopalan, Andrei Maiseyeu

**Affiliations:** 1 School of Medicine, Cardiovascular Research Institute, Case Western Reserve University, 10900 Euclid Ave, Cleveland, OH 44106 USA; 2 Environmental Health and Engineering, Johns Hopkins Bloomberg School of Public Health, Johns Hopkins University, Baltimore, MD USA; 3 Department of Nutrition, Department of Electrical Engineering and Computer Science, Center for Proteomics and Bioinformatics, Case Western Reserve University, 10900 Euclid Ave, Cleveland, OH 44106 USA

**Keywords:** Drug delivery, Obesity

## Abstract

Biodegradable materials, including the widely used poly (lactic-co-glycolic acid) (PLGA) nanoparticles contained in slow-release drug formulations, scaffolds and implants, are ubiquitous in modern biomedicine and are considered inert or capable of being metabolized through intermediates such as lactate. However, in the presence of metabolic stress, such as in obesity, the resulting degradation products may play a detrimental role, which is still not well understood. We evaluated the effect of intravenously-administered PLGA nanoparticles on the gut-liver axis under conditions of caloric excess in C57BL/6 mice. Our results show that PLGA nanoparticles accumulate and cause gut acidification in the cecum, accompanied by significant changes in the microbiome, with a marked decrease of Firmicutes and Bacteroidetes. This was associated with transcriptomic reprogramming in the liver, with a downregulation of mitochondrial function, and an increase in key enzymatic, inflammation and cell activation pathways. No changes were observed in systemic inflammation. Metagenome analysis coupled with publicly available microarray data suggested a mechanism of impaired PLGA degradation and intestinal acidification confirming an important enterohepatic axis of metabolite-microbiome interaction resulting in maintenance of metabolic homeostasis. Thus, our results have important implications for the investigation of PLGA use in metabolically-compromised clinical and experimental settings.

## Introduction

Poly (lactic-*co*-glycolic acid) (PLGA) is one of the most common biodegradable, FDA-approved polymers used in modern medicine and has enjoyed a long history of use as a scaffold/carrier for injectable drugs, vaccines and implantable devices^1^. In the exponentially developing field of drug delivery, nanoparticles (NPs) made of PLGA are a delivery vehicle of first choice, owing to their good biocompatibility, stability, increased safety and modifiable biodegradation rates, thus reducing dosage and potential drug toxicity. Thus, the use of PLGA nanoparticles is of great interest in the design of new drug delivery systems and medical devices^2^; however, there is still knowledge lacking regarding some of their potential effects. For example, it is not completely understood how injected nanoparticle carriers, including PLGA, are sequestered by immune cells, which have enhanced affinity towards particles, and whether their further disposal has an impact on the reticuloendothelial or digestive system. Furthermore, it is still unclear whether certain underlying conditions can have an influence on the response to PLGA.

The interaction between gut microbiota and drug metabolism is still largely unknown^3,4^, albeit an important field of study at present, as it is considered that drug use can be one of the strongest predictors of gut microbiota composition^5^. In this sense, nanoparticles reaching the gastrointestinal tract (GIT) could be having important effects on gut microbiota^6,7^; moreover, gut microbiota may in turn have an effect on the therapeutic outcome of the nanoparticle^7,8^, yet data is still scarce and the mechanisms of action are poorly understood. To date, most work has focused on orally ingested NPs, showing that intake can induce dysbiosis by inhibiting or killing specific microbial members; however, it has been previously shown that intravenous (IV) drugs can also interact with gut microbiota, such as certain IV chemotherapeutic agents^9^. This interaction can lead to severe side effects, warranting the need to further investigate the impact of IV drug delivery systems on the GIT and thus improve their use in treatment. A known example is irinotecan, an IV drug used in colorectal cancer therapy, which is processed in the liver and its by-products transported to the intestine, where it is seen to strongly induce diarrhea^10^. Up until now, studies regarding the impact of injected NPs (PLGA and others) have been mostly carried out in wild-type mouse models or using PLGA particles loaded with drugs or immunomodulators^11^. However, considering the prevalence (and rise) of obesity and the metabolic syndrome^12^, it seems appropriate to broaden the scope and understand the potential impact of these conditions towards IV-administered PLGA.

Studies regarding the effect of PLGA NPs (or any PLGA scaffolds) and their degradation products on gut microbiota are lacking, as well as their potential off-target impact (such as in the liver). Considering that changes in gut microbiota can impact body homeostasis, and that gut microbiota itself may be determining the effect of nanoparticles, it seems necessary to broaden the safety tests carried out so far on PLGA, which have mainly focused on cells and tissues, and have not considered the potential effects on host microbiota^5^. Thus, the purpose of this study was to characterize the effects of repeated administration of intravenous PLGA NPs in an obesity mouse model in the gut and liver, by focusing on gut microbiota composition and gut acidification in particular.

## Results

### Intravenously-delivered PLGA NPs accumulate in the lower GIT through enterohepatic circulation

**Figure 1:**
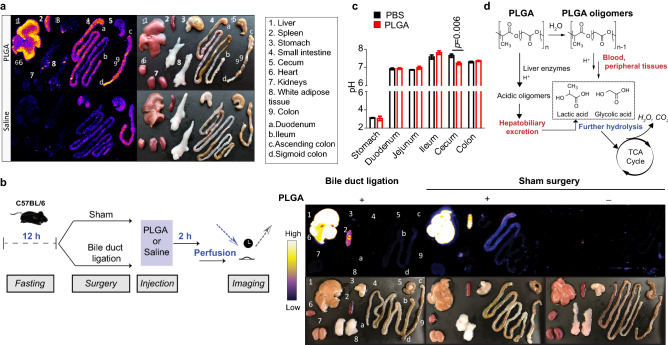
Nanoparticle fate after IV injection. (**a**) Intravenous delivery of a single dose of PLGA-Eu nanoparticles showed their accumulation in major reticuloendothelial organs and the intestinal system, as seen by europium time-resolved fluorescence imaging 4 h post injection. The highest signal was recorded in liver, small intestine and sigmoid colon. Saline-injected mice displayed negligible auto-fluorescence in these organs; (**b**) Bile duct ligation in C57BL/6 mice allowed to determine that PLGA accumulation observed in the lower gastrointestinal tract is due to hepatobiliary excretion into the small intestine, thus it is reabsorbed via the enterohepatic axis; (**c**) C57BL/6 mice (n = 5–7) were fasted overnight (12 h) followed by an IV injection of either PLGA NPs or saline. After 12 h, pH of gastrointestinal sections was measured and cecum pH was found to be significantly lower in PLGA mice compared to PBS (controls) (*t-value*: 3.496; *degrees of freedom*: 10). Independent *t*-test, *p* < 0.05; (**d**) Proposed routes of excretion and metabolism of PLGA degradation products.

It has been previously shown that IV injection of PLGA NPs accumulate in the liver, spleen, lungs, heart and kidneys^13^; furthermore, studies have also shown that orally ingested PLGA NPs are found in the intestine^14,15^. However, to our knowledge, no study has reported whether IV PLGA NPs or their degradation products reach the lower GIT. In order to reliably determine their fate, we synthesized europium-labeled PLGA NPs (PLGA-Eu) in ~ 100 nm size, which is a typical nanoparticle size used in drug delivery (Supplementary Fig. 1). A single injected dose of PLGA-Eu in C57BL/6 mice accumulated in the liver and to a lesser degree in the spleen; however, more interestingly, high amounts were also observed in the small and large intestine, notably in the sigmoid colon (Fig. 1A). These results led us to hypothesize that PLGA NPs travelled to the GIT via enterohepatic circulation; thus, for this, we performed an acute experiment of bile duct ligation in C57BL/6 mice followed by PLGA-Eu injection (Fig. 1B). Imaging of excised organs at the end of the experiment allowed us to observe that PLGA NPs were only found in the liver and spleen, therefore confirming the involvement of the enterohepatic axis in PLGA disposal. In all, we were able to show for the first time that these nanoparticles accumulate to a significant level in the lower GIT when delivered intravenously through hepatobiliary excretion into the small intestine.

### IV-injected PLGA NPs reach the cecum and lower fecal pH

After observing that PLGA reaches the lower GIT in a significant manner, and considering that PLGA biodegradation yields the formation of glycolic and lactic acid, we hypothesized that these by-products could influence gut pH. Thus, we administered a single dose of partially degraded PLGA NPs to C57BL/6 mice via oral gavage or IV and observed that they were able to significantly lower pH in colon when administered orally, and in cecum when injected IV (Supplementary Fig. 2). In order to confirm these results, C57BL/6 mice were fasted overnight followed by an IV injection of either PLGA NPs or saline. After a single IV injection of PLGA NPs, pH was significantly lower in the cecum (Fig. 1C), suggesting that gut acidification is likely driven by the excretion and metabolism of PLGA degradation products (acidic oligomers, lactic acid and glycolic acid) (Fig. 1D).

### Repeated administration of IV PLGA NPs does not affect body weight or body fat accumulation in C57BL/6 obese mice

**Figure 2:**
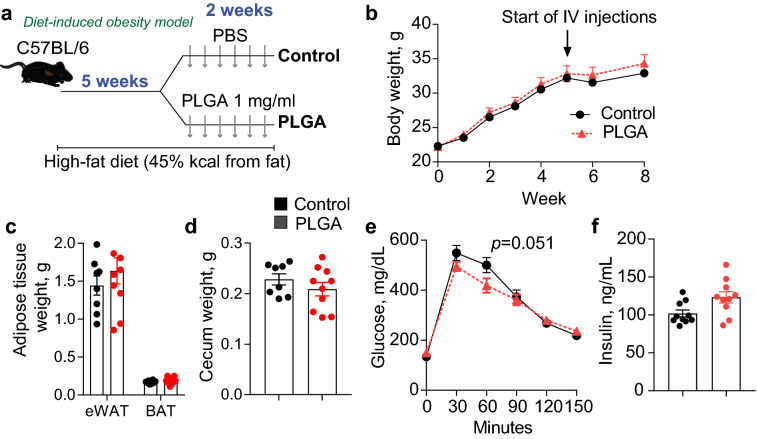
Effect of PLGA on body weight and glucose metabolism in diet-induced obese mice. (**a**) C57BL/6 mice were fed a high-fat diet for 5 weeks and were then injected IV with either PLGA nanoparticles or PBS six times during two weeks; (**b**) Body weight throughout experiment and (**c**) adipose tissue and (**d**) cecum weight at the end of the study were not significantly altered by treatment; (**e**) IPGTT before euthanasia revealed that PLGA nanoparticle-injected animals presented significantly better glucose clearance at 60 min (n = 10 mice/group); (**f**) Fasting insulin was not different between groups. n = 10 mice/group, independent *t*-test, *p* < 0.05.

The traditional view of lactate as a major product of anaerobic glycolysis and ATP production under strict homeostatic control^16^ has now been replaced by an expansive concept that it is a nutrient in almost every major organ, potentially serving as an essential signaling molecule and acting as the primary source of energy in the TCA cycle^17–19^. Taking into consideration the fact that PLGA produces lactate during its biodegradation, it seemed relevant to determine whether the administration of this nanoparticle could be providing excess fuel and thus have an impact on body weight. For this, we conducted a first experiment in which normal weight, chow-fed C57BL/6 mice were injected with PLGA NPs (NW-PLGA) or saline (NW-PBS) a total of six times in a 2-week period, monitoring body weight throughout. By the end of the experiment, we determined that nanoparticle treatment did not alter body weight (Supplementary Fig. 3A). We then hypothesized that in an altered metabolic state, such as obesity, repeated administration of PLGA could have an impact on overall body weight and fat accumulation. Thus, C57BL/6 mice were fed a high-fat diet for 5 weeks in order to induce obesity, followed by the repeated IV injection of PLGA NPs or saline (controls) as described above (Fig. 2A). We found that nanoparticle treatment did not significantly alter body weight compared to controls (Fig. 2B), body fat mass (epididymal white adipose tissue and brown adipose tissue) (Fig. 2C), cecum (Fig. 2D) or liver, heart, skeletal muscle, and kidneys (data not shown). This lack of differences associated to PLGA treatment is consistent with previous studies using similar (*ob/ob*) models^14^.

### PLGA NPs alter glucose homeostasis in obesity

When PLGA undergoes hydrolytic degradation (Fig. 1D), lactic and glycolic acids are produced, both known substrates for gluconeogenesis in liver. Considering sustained excessive hepatic glucose production can contribute to hyperglycemia and type 2 diabetes, we investigated whether prolonged IV PLGA NP administration has an impact on hyperglycemia. For this, we carried out an intraperitoneal glucose tolerance test (IPGTT) at the start of delivery, after one week of injections and at the end of the two-week treatment in both normal weight, chow-fed C57BL/6 mice and diet-induced obese mice. IV injection of PLGA did not have an effect on glucose clearance in normal weight mice (Supplementary Fig. 3B). However, when carrying out the same experiment in diet-induced obese animals (Fig. 2A), PLGA-treated mice showed a slight improvement in glucose clearance 60 min after intraperitoneal (IP) injection of glucose (*p* = 0.051) at the end of treatment (Fig. 2E), yet recovery levels were the same for both groups (PLGA vs. control). Furthermore, no changes were observed regarding insulin levels (Fig. 2F).

### IV PLGA impairs intracellular glucose uptake and lowers plasma lactate levels

**Figure 3:**
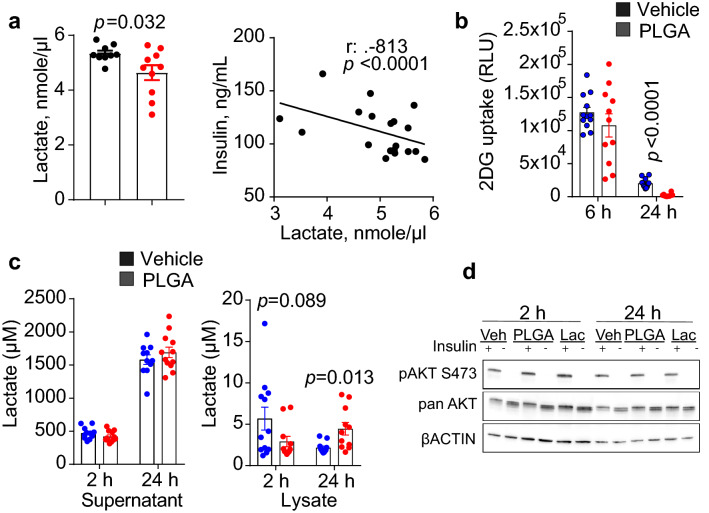
PLGA alters intracellular glucose uptake and lowers lactate levels. (**a**) Administration of PLGA nanoparticles for 2 weeks significantly lowered plasma lactate levels (*t-value*: 2.435; *degrees of freedom*: 11.528) (n = 10 mice/group). Furthermore, plasma lactate was negatively correlated with plasma insulin levels; (**b**) 2-deoxy-D-( +)-glucose (2DG) uptake in L6 myotube cells was significantly lower after 24 h treatment with PLGA (1 mg/mL) (*t-value*: 8.359; *degrees of freedom*: 13.698) (n = 12 replicates/group); (**c**) Lactate concentrations in supernatants and lysates of L6 myotubes were significantly higher in cell lysates after 24 h treatment with PLGA (1 mg/mL) (*t-value*: − 2.942; *degrees of freedom*: 11.476) (n = 12 replicates/group); (**d**) Insulin signaling experiments in L6 myotubes treated with 1 mg/mL PLGA, 50 mM lactate (Lac) or vehicle control (veh) showed no difference in levels of phosphorylated AKT in response to 100 nM insulin for 5 min. Representative images of phosphorylation on AKT residue Ser^473^ and loading controls, pan AKT and βACTIN are shown. Uncropped blot images are presented in supplementary files. Independent *t*-test was used when comparing two groups and correlation was determined by Spearman’s rank correlation analysis, *p* < 0.05.

Contrary to what was expected, plasma lactate concentrations in PLGA-injected obese mice were significantly lower than controls (Fig. 3A), and were negatively correlated with plasma insulin. Because of this finding and the fact that fasting glucose levels did not change between groups, we hypothesized that PLGA NPs may have an effect on glucose uptake and metabolism in skeletal muscle, the largest lactate producing organ. In order to study this, we conducted glucose uptake experiments in differentiated rat L6 myotubes, a well-characterized model of skeletal muscle cells, showing that the uptake of 2-deoxy-D-glucose (2DG) in rat L6 myotubes was diminished at 6 h and was significantly lower following 24 h treatment with PLGA NPs as compared to control (Fig. 3B). In line with this finding, lactate release in the supernatants of L6 myotubes was reduced at 2 h of treatment, however no significant difference was observed after a 24 h incubation with PLGA NPs. Intracellular lactate (L6 cell lysates) was lower in PLGA NPs treated cells at 2 h, yet significantly higher at 24 h (Fig. 3C). Collectively, this data suggest that glucose uptake in PLGA NPs treated muscle cells is impaired early on after PLGA NPs exposure, and that cells accumulate intracellular lactate, possibly due to defective lactate efflux. Because AKT signaling is central to downstream effects of insulin signaling^20^, we also tested levels of AKT phosphorylated at serine 473 (pAKT^S473^) of cells treated with PLGA NPs in response to insulin stimulation. However, immunoblotting experiments in L6 myotubes (Fig. 3D) demonstrated that phosphorylation of AKT^S473^ by insulin was unaffected by PLGA treatment (2 h and 24 h). Although warranting further investigation, the effect of PLGA NPs on lowering plasma lactate levels was likely due to a decrease in anaerobic glycolysis in muscle and/or defective monocarboxylate transporters (such as MCT1)^19^, but not secondary to an effect on insulin resistance. Thus, the actions of PLGA in skeletal muscle are intriguing, however beyond the scope of this manuscript.

### Intravenous PLGA NPs do not have an effect on plasma lipids, cytokines and LPS levels in obese mice

Obesity is characterized by an inflammatory state, with significantly altered plasma lipid profiles and increased cytokine levels^21^. In order to understand whether PLGA NPs could be interfering with such metabolic inflammation, we analyzed the levels of a wide range of pro- and anti-inflammatory mediators and chemokines in plasma samples from obese mice injected with PLGA NPs. We found that nanoparticle treatment significantly decreased IL-4 compared to controls (Table 1); however, no other cytokines were altered. On the other hand, we also tested plasma levels of lipopolysaccharide (LPS), which is produced by gram-negative bacteria in the gut and released into the bloodstream, potentially leading to low-grade inflammation^22^, and is ultimately considered a marker of metabolic endotoxemia. No changes in plasma LPS concentration were observed when comparing both groups (Table 1) which, together with the cytokine data, indicates that two weeks of PLGA NP administration does not induce, or exacerbate, systemic inflammation. Furthermore, PLGA NPs did not have an effect on plasma cholesterol and triglyceride (TG) levels in these mice, suggesting that hepatic lipid homeostasis was unaffected.

**Table 1:** Plasma levels of lipids, inflammatory cytokines and LPS.

	Control	PLGA	*P* value
**Cytokines, pg/mL**			
*CXCL1/KC*	91.04 ± 8.42	98.47 ± 14.83	*0.668*
*CXCL5*	968.76 ± 173.21	1,172.98 ± 208.47	*0.477*
*IL1ɑ*	44.86 ± 17.27	17.54 ± 3.19	*0.156*
*IL2*	10.24 ± 1.59	13.86 ± 1.71	*0.142*
*IL4*	98.45 ± 2.46	91.22 ± 2.15	0.040*
*IL5*	3.89 ± .8.03	5.64 ± 2.05	*0.446*
*IL13*	237.11 ± 5.91	224.69 ± 10.04	*0.319*
*MCP-1*	46.06 ± 7.24	46.69 ± 12.37	*0.964*
*MIP-2*	99.22 ± 9.76	77.10 ± 13.03	*0.190*
**Plasma endotoxin (EU/mL)**			
*LPS*	3,720.00 ± 83.69	3,718.50 ± 110.17	*0.991*
**Plasma lipids, mg/dL**			
*Triglycerides*	79.42 ± 2.70	89.26 ± 4.67	*0.95*
*Cholesterol*	106.74 ± 4.77	100.14 ± 6.88	*0.46*

*CXCL1/KC* chemokine (C-X-C motif) ligand 1, *CXCL5* chemokine (C-X-C motif) ligand 5, *IL* interleukin, *LPS* lipopolysaccharide, *MCP-1* monocyte chemoattractant protein 1, *MIP-2* macrophage inflammatory protein 2.

*p < 0.05.

### Intravenous PLGA NPs alter gut microbiota composition in obese mice

It is well documented that gut microbiota is altered in obese states^23^; thus, following the observation that IV PLGA nanoparticles reach the cecum and cause a significant acidification of its contents, it was of interest to determine whether it could influence gut microbiota composition. For this, after treating C57BL/6 obese mice with PLGA NPs for 2 weeks, cecum feces were collected for 16S gene amplification to characterize phylogenetic variation at different taxonomic levels.

**Figure 4:**
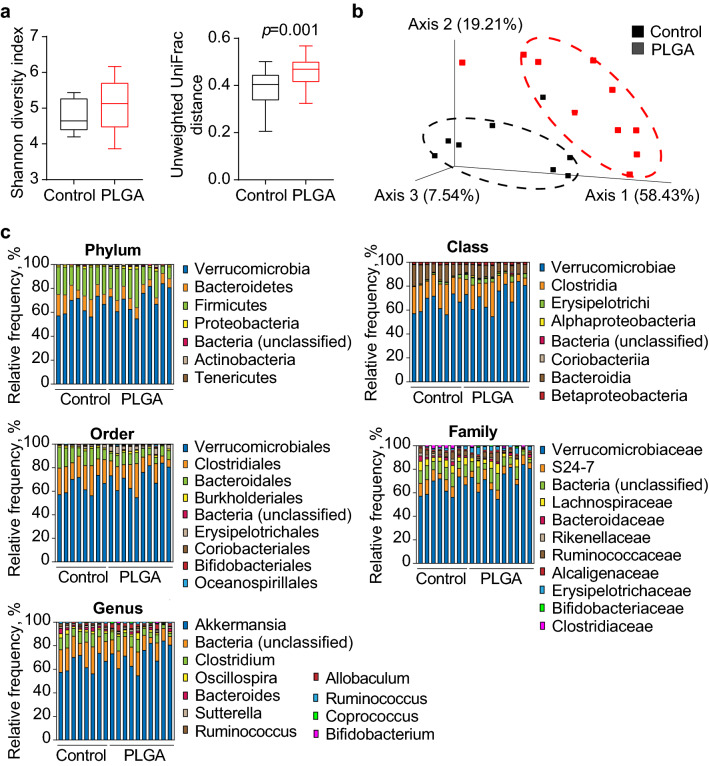
Bacterial diversity measurements show that gut microbiota diversity was affected by treatment. (**a**) Alpha diversity was determined using the Shannon diversity index on raw OTU abundance after filtering out contaminants (not significant, *p* = 0.286). However, when comparing phylogenetic tree information between groups using the unweighted unique fraction (UniFrac) distance measurement, there was a significant difference regarding gut microbiota diversity (pairwise Permanova) (n = 10/group); (**b**) Gut microbiota composition similarity among groups was represented using a principal coordinate ordination, based on weighted UniFrac distances, where points are individual samples; (**c**) Stacked column graphs show the relative frequency of bacterial species in control and PLGA mice in the gut microbiota of cecum feces, analyzed using Qiime2 Naive Bayes classifier using Greengenes (v13.5). Statistics: n = 10 mice/group, pairwise Kruskal–Wallis test (when comparing diversity indices), *p* < 0.05.

Community richness and diversity measurements between control and PLGA NPs-treated mice were carried out in order to determine the impact of IV PLGA NPs had on cecum microbiota. The Shannon diversity index (α-diversity index), which defines the richness and evenness within a microbial community^24^, indicated no major shifts (*p* = 0.286) (Fig. 4A). However, the unweighted unique fraction (UniFrac), which indicates the phylogenetic distance between taxa in a phylogenetic tree^25^, was significantly different between groups, suggesting a change in microbial composition and phylogenetic distance due to PLGA NPs (Fig. 4A).

In order to further study similarity (or dissimilarity) in terms of microbiota composition among samples, data was submitted to a principal coordinate ordination based on weighted UniFrac distances (Fig. 4B). As observed in the scatter plot, axis 1 represents 58.43%, axis 2 represents 19.21% and axis 3 represents 7.54% diversity as a percentage, in which the two groups clustered separately, suggesting a clear difference in community structure of gut microbiota driven by PLGA NPs administration under high-fat feeding conditions. In order to understand the effects of PLGA on specific bacterial communities, sequences were classified into operational taxonomic units (OTUs) and categorized by individual microbial taxa (Fig. 4C), including phylum, class, order, family and genus clusters. The gram-positive Firmicutes and gram-negative Bacteroidetes phyla are the most predominant (comprising approximately 90% of gut microbiota) and studied bacterial phyla^26,27^. Both were seen to be affected by treatment and were downregulated in PLGA NPs-treated mice in a statistically significant manner (as per analysis of composition of microbiomes (ANCOM), see Supplementary Table 1 for detailed results). To our knowledge, this is the first study that has specifically shown that IV PLGA NPs can alter gut microbiota in an obese mouse model; however, further studies are needed in order to determine the impact of these changes.

### Predicted metagenomic pathways indicate PLGA NPs affect those associated to metabolism

**Figure 5:**
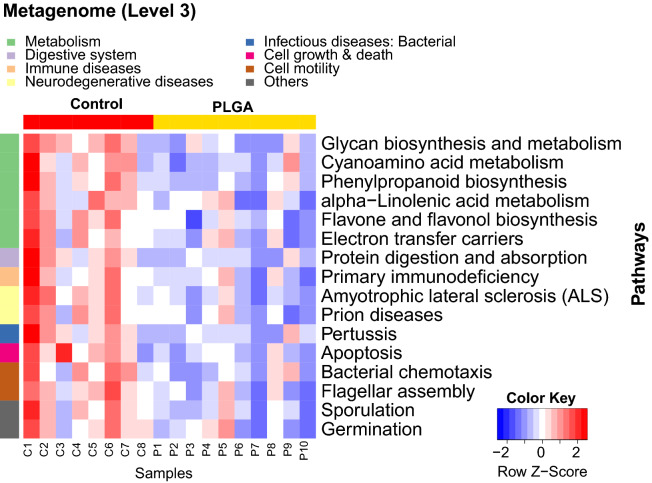
Metagenome pathway analysis predicts metabolism aberrations due to PLGA treatment. The functional composition of metagenomes was predicted with PICRUSt (phylogenetic investigation of communities by reconstruction of unobserved states). These gene mappings were then used to reconstruct metagenomic pathways. The gene content estimations were mapped to KEGG pathways^15^ to identify the functional enrichment of these pathways, and the Wilcoxon Rank Sum test was used to determine significant differences (adjusting with Bonferroni correction). The heatmap depicts the significant differences in pathway enrichment (*p* < 0.05), where upregulation is indicated in red and downregulation in blue. Controls (red) vs. PLGA (yellow) samples are shown at the bottom of the heatmap, and pathways affected are organized according to KEGG pathway categories. N = 10/group.

Bacterial sequence reads of both controls and nanoparticle-treated mice were mapped to Kyoto Encyclopedia of Genes and Genomes (KEGG) reference pathways to predict the functional composition of metagenomes with PICRUSt. Interestingly, cell motility (*p* = 0.020) and digestive system (*p* = 0.006) pathways were significantly downregulated in animals who received PLGA NPs (data not shown). At a further level (Fig. 5), analysis indicated that a total of 16 pathways were statistically significant (*p* < 0.05) between PLGA and control mice, and nearly half of these were associated to metabolism functions. Carbohydrate, protein, and lipid-related pathways were downregulated in PLGA NPs-treated animals, as well as the electron transfer carrier pathway. Collectively, this suggests that PLGA NPs may have an effect on systemic metabolism, including oxidative phosphorylation and mitochondrial function.

### Whole transcriptome analysis of hepatic gene expression

**Figure 6:**
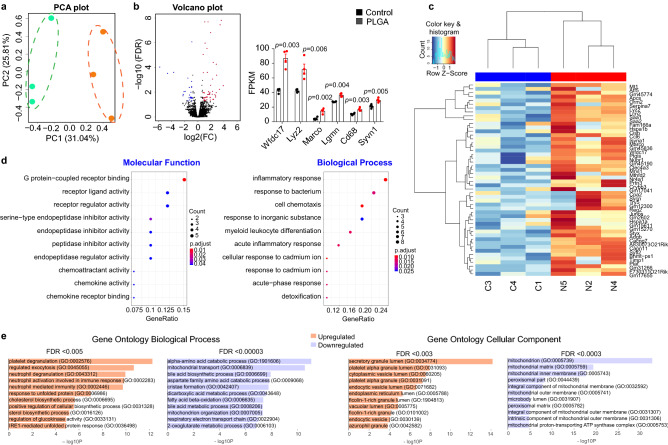
RNA-seq transcriptome analysis identifies metabolism, enzymatic degradation and cellular stress pathways in liver. (**a**) Principal component analysis (PCA) of whole-transcriptome RNA-seq read counts in liver. Dotted ellipses indicate the 95% confidence interval of samples that fall into two distinct groups (PLGA nanoparticle-treated and control). Axis percentages indicate variance in the data contribution (n = 3/group); (**b**) Volcano plot indicating the genes in liver with significantly increased (red dots) or decreased (blue dots) expression in PLGA treated group compared to control. The x-axis shows log2 fold-changes (FC) in the expression and the y-axis the log 10 false discovery rate (FDR) of a given gene being differentially expressed. Selected most significantly regulated genes are plotted in the bar graph as gene vs. fragments per kilobase of transcript per million mapped reads (FPKM): Wfdc17: activated macrophage/microglia WAP domain protein (*t-value*: − 7.225; *degrees of confidence*: 3.414); Lyz2: lysozyme C-2 (*t-value*: − 4.166; *degrees of confidence*: 6); Marco: macrophage receptor with collagenous structure (*t-value*: − 5.416; *degrees of confidence*: 6); Lgmn: legumain (*t-value*: − 7.369; *degrees of confidence*: 3.283); CD68: cluster of differentiation 68 (*t-value*: − 4.777; *degrees of confidence*: 6); Syvn1: Synoviolin 1 (*t-value*: − 4.286; *degrees of confidence*: 6); (n = 4 mice/group, independent *t*-test, *p* < 0.05); (**c**) Heatmap of hierarchical clustering indicates differentially expressed genes (columns) in liver from individual control (C3, C4, C1) and PLGA nanoparticle-treated animals (N5, N2, N4) samples (n = 3/group); (**d**) Gene ontology (GO) analysis presented as a scattergram of overrepresented GO terms in molecular function and biological process categories; (**e**) Additional GO analysis using more stringent FDR filtering (as indicated above the plot) demonstrated upregulation of various cell exocytosis and secretion pathways and downregulation of oxidative metabolism (mitochondrial function) pathways in liver.

After intravenous injection, PLGA NPs are released into the bloodstream and are sequestered by the liver^28^, and the biological processes activated in response to this filtration may be characteristic of defense/clearance mechanisms against a nanoparticle challenge. In an attempt to understand the effects of PLGA administration in liver, we analyzed the hepatic response at the transcriptome level by using an unbiased approach. This type of analysis, to our knowledge, has not been previously published to date. Thus, RNA sequencing (RNAseq) of the whole liver was carried out to determine potential changes induced by PLGA nanoparticle treatment, showing a significant increase in the expression of 41 genes and a decreased expression of 21 genes (Supplementary Table 2). There was a significant treatment effect as per principal component analysis (Fig. 6A), with some genes changing expression level by more than eight-fold (Fig. 6B). Notably, a number of upregulated genes was associated with macrophage/monocyte-specific markers, such as lysozyme C-2 (Lyz2), macrophage receptor with collagenous structure (Marco) and cluster of differentiation 68 (CD68), as well as with immune cell activation, including activated macrophage/microglia WAP domain protein (Wfdc17). Hierarchical clustering analysis (Fig. 6C) showed that the gene expression patterns between control and PLGA NPs-treated animals are highly dissimilar, further confirming the PLGA NP effect.

The analysis of transcriptional differences via gene ontology (GO) enrichment pathways (Fig. 6D,E) revealed that the most upregulated “Biological Process” pathways were exocytosis/endocytosis (platelet degranulation, regulated exocytosis). Interestingly, at least two sterol biosynthetic processes were upregulated; yet, as we showed above, plasma cholesterol levels were unchanged (Table 1). Remarkably, GO terms such as mitochondrial transport and respiratory electron transport chain (among others) were significantly downregulated, indicating diminished mitochondrial function and oxidative phosphorylation. On the other hand, upregulated GO terms included exocytotic pathways, likely because of PLGA nanoparticle engulfment and exocytosis of unprocessed PLGA oligomers, as well as enzyme activity, inflammatory response and cellular stress (Fig. 6D).

Next, differentially-regulated genes were annotated with the KEGG pathway database (Supplementary Fig. 4). We found that significantly overrepresented upregulated GO terms were related to cellular processing (protein processing endoplasmic reticulum, lysosomes) while downregulated terms included metabolic pathways, carbon metabolism and the TCA cycle. Taken together, these data suggest that PLGA NPs significantly change metabolism in liver under high-fat feeding conditions by altering mitochondrial activity, inflicting cellular stress and upregulating pathways needed for exocytotic removal.

### Analysis of PLGA-relevant transcriptional pathways in human GIT

**Figure 7:**
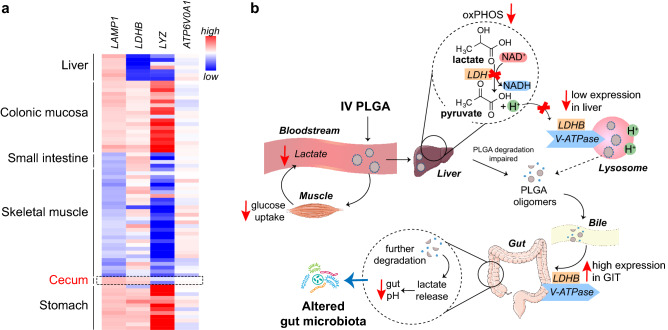
Microarray data mining suggests possible PLGA nanoparticle degradation pathways in liver and GIT. (**a**) The expression of lysosome-associated genes (LAMP1, LYZ), vacuolar type H^+^-ATPase (ATP6V0A1) and lactate dehydrogenase B (LDHB) was determined through the MERAV database (see “Methods”). Heatmap represents the expression levels of the indicated genes in tissues hypothesized to be involved in PLGA nanoparticle degradation and disposal; (**b**) Proposed degradation pathways of PLGA nanoparticles in various organs and compartments (see main text). *IV* intravenous, *LDH* lactate dehydrogenase, *NAD*^*+*^ nicotinamide adenine dinucleotide oxidized form, *NADH* reduced form of NAD, *oxPHOS* mitochondrial oxidative phosphorylation, *GIT* gastrointestinal tract.

The enterohepatic circulation is a major route of metabolism and refers to the transportation of many drugs from the liver to the small intestine, where they are absorbed, metabolized and, depending on the metabolite, transported back to the liver^29^. Given our RNAseq data and the fact that nanoparticles are well-known to break down intracellularly through lysosomal catabolic degradation, we used the Metabolic Gene Rapid Visualizer (MeRav) database^30^ to search for lysosome-related mRNAs co-expressed in human tissues (Fig. 7A and Supplementary Fig. 5A, Supplementary Table 3) and undertook this analysis in various organs involved in lactate metabolism. Lactate dehydrogenase B (LDHB) was of particular interest, being an intracellular enzyme catalyzing the interconversion of lactate to pyruvate in an NAD + /NADH-dependent manner (Fig. 7B). Although LDH exists as five different isoenzymes, LDHB isoform is known to have a preference towards lactate-to-pyruvate conversion. Moreover, LDHB was recently shown to play an essential role in lysosomal degradation, interacting with vacuolar type H^+^-ATPase (ATP6V0A1)^31,32^. Interestingly, we found that whereas LDHB is not expressed in liver, it is highly expressed in the GIT, including the cecum (Fig. 7A and Supplementary Fig. 5A). Strikingly, LDHB was co-expressed with ATP6V0A1 and major lysosomal markers such as lysosomal associated membrane protein 1 (LAMP1) and Lyz in GIT, but not in liver or the skeletal muscle. Lastly, ATP6V0A1 expression significantly correlated with the expression of LDHB in these tissues with Pearson coefficient of 0.53 and *p* < 0.0001 (Supplementary Fig. 5B).

## Discussion

PLGA polymers are widely used in medicine, acting in many cases as carriers for certain drugs through intravenous routes. However, even though they are considered safe, there are still gaps in knowledge regarding potential long-term effects, especially in the GIT and liver. In this study, we show that IV PLGA NPs in a murine obesity model reach the lower GIT, likely through enterohepatic circulation, decrease cecum pH and alter gut microbiota composition. Furthermore, RNAseq revealed concomitant changes in hepatic gene expression associated with cellular stress and mitochondrial function, confirming the importance of the enterohepatic axis working in concert particularly in conditions of metabolic stress. Moreover, our results demonstrate that PLGA NPs do not worsen glucose clearance under conditions of caloric excess, and do not increase lactic acid levels systemically or result in hyperinsulinemia. Given the importance of PLGA as a widely used nanoparticle, these results are of considerable importance.

Our first observation was that IV PLGA NPs accumulated in a notable fashion in the lower GIT, whereby nanoparticles travelled from the liver to the gut through enterohepatic circulation. Intestinal elimination of nanoparticles after oral administration has been investigated previously with the help of fluorochrome tags. However, background fluorescence often impedes particle tracking, especially in the GIT where remnants of bile, diet and digestion products may interfere with imaging. In this study, we designed a PLGA NP loaded with an europium tag that circumvented these issues by virtue of time-resolved fluorescence (TRF), which eliminates background fluorescence allowing for reliable imaging. This, coupled with an acute experiment involving bile duct ligation, by which we achieved experimental obstruction of the extrahepatic biliary system, allowed us to determine with a high degree of confidence that PLGA NPs and their degradation products accumulate in the lower GIT via the liver. Since europium complexes are eliminated through glomerular filtration in kidneys^33^, the europium fluorescence signal in the GIT is highly suggestive that at least a part of the PLGA polymer (or a portion of its non-degraded particles) is still attached to the europium label following hepatobiliary excretion. Even though there is currently a wide nano-scale range available, the nanoparticle size (100 nm) chosen in this study is commonly used in drug delivery research; most importantly, many investigators report off-target nanoparticle clearance by liver (> 80% of the injected dose in many cases), which is likely driven by the size of liver sinusoidal endothelial fenestrations, ranging from 100 to 150 nm^34^. Larger particles (> 200 nm), however, are known to be removed by the spleen^35^. Thus, further studies are required to fully understand the impact of various nanoparticle sizes.

The impact of drugs delivered both orally and systemically on the microbiome is starting to gain relevance, as this may not only determine the treatment outcome but may also have off-target effects. However, the impact of many drugs on gut bacterial composition, through either direct or indirect mechanisms, remains unknown^5^. A recent study which screened over 1,000 marketed non-antibiotic drugs found that 24% inhibited the growth of at least one bacterial strain in vitro^37^; in the field of nanoparticles, though, such analysis has yet to be carried out. Data is emerging regarding the effect on gut microbiota of some widely used nanoparticles, including chitosan^38^, silver^39–42^, octahedron iron oxide^43^, TiO2^44,45^, gold^46^, and fullerenol^47^. Furthermore, similar studies looking at the effects of oral nanoparticle formulations have reported small (or no) effects on gut microbiota^39,45,48^. Thus, after our initial observation that IV PLGA reach the lower GIT in a significant manner, we demonstrate that both oral and IV PLGA NPs reduce cecum pH significantly, most likely driven by the formation of glycolic and lactic acid. The density of microbiota increases steadily throughout the GIT, determined in great part by the acidity of each compartment. Thus, the stomach (10^1^ bacteria/g) and duodenum (10^3^ bacteria/g) are highly acidic and have a low concentration of bacteria compared to the cecum (10^12^ bacteria/g), characterized by a more neutral pH^36^. Considering that pH greatly affects bacteria abundance and diversity, any significant drug-induced changes on intestinal pH will most likely have an impact on gut microbiota and should be considered, since growth (or reduction) of certain bacterial species could compromise intestinal mucosa and/or function. In this sense, we report significant differences regarding bacterial diversity and community structure between groups, whereby PLGA nanoparticle treatment decreased both Bacteroidetes and Firmicutes significantly. Thus, the fact that an IV-administered nanoparticle formulation was even capable of modifying gut microbiota composition is of importance and warrants further research to determine the potential effects, and its biological significance, in the long term. Furthermore, as recently discussed^5^, even though the effect of non-antibiotic drugs on gut microbiota may not result in changes in the therapeutic outcome, it is still essential to understand gut microbiota-drug interactions in order to improve microbial manipulation in health and disease. In the case of PLGA, alteration of phyla such as Bacteroidetes and Firmicutes could be, in part, driving some of the effects here described in liver and other (not studied) organs and tissues. It is important to note, however, that our study was carried out over a 2-week period in an obese murine model and in many cases this formulation is given over a longer period of time due to the nature of the treatments it is used in.

Another key observation in this study is that PLGA NPs caused transcriptomic changes in liver, mostly associated with exocytosis/endocytosis, cell stress and mitochondrial function. In order to link the observed changes with the fact that PLGA NPs degradation in liver was impaired, we employed microarray data mining that identified a possible “natural history” of PLGA disposal. Collectively, our data suggest that the route of PLGA NPs from liver to bile is likely to involve exocytosis of undigested particles, further adsorption in GIT and final degradation in the large intestine, resulting in PLGA-derived lactate release and reduction in pH. Under conditions of caloric excess, the ability of the liver to metabolize carbohydrates, lipids and amino acids could be challenged (Fig. 5), resulting in low availability of NAD equivalents needed for the conversion of PLGA-derived lactate to pyruvate (Fig. 7B). The lack of liver expression of LDHB, involved in lysosomal degradation, may play a role in incomplete PLGA processing in this organ, resulting in a dependence on the gut and other organs for the efficient disposal of lactic acid. In this sense, the expression of vacuolar type H^+^-ATPase (ATP6V0A1), a crucially important enzyme that acidifies lysosomal compartment and may facilitate PLGA hydrolysis, was highly correlated with LDHB and co-expressed only in GIT. Together, these data suggest that efficient PLGA degradation requires a concerted coupling of both LDHB and ATP6V0A1, likely in the gut; however, further extended investigation is needed to fully support this hypothesis.

Lastly, it is known that a fraction of the injected nanoparticle is engulfed by innate immune system cells, such as macrophages and monocytes, whereby an estimated 10–40% of an injected dose can accumulate in monocytes and macrophages in blood, spleen and bone marrow^49^. Even though we did not study the amount of PLGA engulfed by these cells, we observed a significant upregulation of monocyte-specific markers in the liver of PLGA-injected animals, as seen in RNAseq analysis. More specifically, Lyz2 was highly upregulated in the PLGA treatment group (Fig. 6B,C), which is indicative of liver-infiltrating blood monocytes^50^, which. Similarly, a newly-discovered marker of circulating inflammatory and patrolling monocytes, C-type lectin domain family 4, member a3 (Clec4a3)^51^, was strongly upregulated in PLGA mice (Supplementary Table 2). Furthermore, chemoattractant activity GO terms were also upregulated in PLGA-treated animals (Fig. 6D), further confirming the possibility of the migration of monocytes that engulfed PLGA. Collectively, this suggests that PLGA-laden inflammatory monocytes from circulation and bone marrow may migrate and accumulate in the liver, especially in conditions of developing metabolic disease^50^.

Thus, in this study we report an unexpected effect of intravenous PLGA in mice under a high-fat diet, including gut microbiota alteration coupled with coordinate transcriptomic enterohepatic reprogramming, likely through changes in intestinal pH, yet without a significant impact on glycemia or insulin levels. Given the importance of PLGA NPs and their widespread use, we consider these results of relevance for future work regarding PLGA nanoparticle use in metabolically-compromised clinical and experimental settings.

## Methods

### PLGA NP synthesis and delivery

The dose of PLGA for in vivo experiments was selected based on maximal achievable concentration of PLGA in the solution (10 mg/mL), at which PLGA is stable in PBS and does not precipitate for at least 24 h after synthesis. The injection volume was selected based on maximal injection volume in mice as recommended previously^52^. Europium cryptate was included in the PLGA formulation only when performing imaging experiments. For chronic experiments in mice, europium was omitted from the formulation to exclude possible confounding effects.

PLGA polymers (described in Supplementary Table 4) were dissolved in acetonitrile at 10 mg/mL with the following ratio (mass by volume): 20% mPEG-bPLGA: 75% PLGA (Resomer RG 502 H): 1% PLGA-NH-Eu (see below). This acetonitrile solution was added dropwise via a syringe pump to vigorously stirred water (1,000 rpm) at a rate of 4 mL/h. The final acetonitrile concentration in water was set to not exceed 10%. The resulting solution was then transferred to Amicon Ultra-15 centrifugal unit (Millipore-Sigma, cat# UFC903024) and spun down for 10 min × 2,000*g*. The NPs were then washed with ultrapure water three times on the same centrifugal unit (each 10 min × 2,000*g*) followed by concentration down to approximately 10–20 mg/mL based on initial total polymer weight. The NPs were collected and a 0.1 mL portion of the solution was then evaporated in a pre-weighted tube using a vacuum concentrator (speedvac). The weight of the tube with the solid polymer was recorded using 6-place balance (Mettler Toledo XPR, precision 0.000001 g) and PLGA concentration was determined. Aqueous solutions of PLGA NPs were used for tail vein injection, adjusting them to physiological tonicity by addition of 1 M HEPES buffered saline (pH 7.4) to a final concentration of 50 mM of HEPES and 154 mM sodium chloride.

PLGA-Europium-Cryptate (PLGA-Eu) was made in house from europium cryptate (trFluor Eu-Cryptate succinimidyl ester, AAT Bioquest, Inc. Sunnyvale, CA) (1.3 equivalent, 1.3 µmol, 1 mg) that was added to a dry reaction vessel, following which 36 mg (1.25 µmol) of PLGA-NH2 (MW 28 kDa, PolySciTech, cat# Al10) was then added in 0.5 mL dichloromethane, plus 2 µL of dry triethylamine and the mixture was stirred at room temperature for 18 h. The solution was evaporated under nitrogen gas and dissolved in acetonitrile at 1 mg/mL. The conjugate was stored at − 20 °C until use.

### Animals

Male, C57BL/6 mice (5 weeks of age) were purchased from The Jackson Laboratory (Bar Harbor, ME) and kept in AAALAC-accredited facilities at Case Western Reserve University. Animals were housed five per cage and allowed to acclimate in the facility for one week. Throughout the experiments, animals were kept on a 12:12 h light–dark cycle at 22 °C, and both diet and water were provided ad libitum. The protocols followed and the use of animals here described were approved by and in accordance with The Institutional Animal Care and Use Committee (IACUC) (protocol number: 2016-0273, Case Western Reserve University). All experimental procedures were conducted following the IACUC guidelines and regulations.

### Experimental design

An initial, preliminary study was carried out in male C57BL/6 mice, which were normal weight and fed a standard chow diet. Animals were injected with 30 mg/kg PLGA in 100 µL (n = 6) (NW-PLGA) or a saline solution (physiological 0.9% sodium chloride) (NW-PBS) (n = 4) a total of six times in a 2-week period (3 times/week).

For the following study, all animals were placed on a high-fat (45% kcal from fat) diet (D12451, Research Diets, New Brunswick, NJ) and were fed for five weeks in order to establish obesity and onset of insulin resistance. This was then followed by randomization and treatment, where half of mice (n = 10, PLGA) received PLGA nanoparticles via tail-vein injection at 30 mg/kg (in 100 µL) three times a week for two weeks, whereas the other half (n = 10, Control) received a saline infusion (physiological 0.9% sodium chloride). All mice remained on the same high-fat diet throughout the treatment period.

### Time-resolved fluorescence imaging of excised organs

Two weeks before PLGA or saline injection and imaging of mice, animals were switched to a low-fluorescence, alfalfa-free diet (Research Diets, TD.97184 (purified)), provided ad libitum. Mice (n = 1/group) were fasted overnight (12–14 h) and then injected through a penile vein with 30 mg/kg of PLGA-Eu. Four hours later, mice were euthanized and organs depicted in Fig. 1 were extracted. The organs were positioned on a low-fluorescence mat that was placed on a 96-well plate insert of Molecular Devices i3 plate reader with a TRF module (WB cartridge) and the plate was imaged using excitation of 340 nm and emission 615 nm. Large Stokes shift of europium coupled with TRF tracking allowed for minimal background auto-fluorescence and high signal-to-noise ratio imaging of PLGA disposal in the liver and throughout the digestive system. Images were analyzed using Molecular Devices SoftMax Pro 6.2 software.

### Bile duct ligation

C57BL/6 male mice (age = 9 weeks) were fasted for 12 h prior to the intervention. First, animals were anesthetized with 100 mg of ketamine/kg body weight and 10 mg of xylazine/kg body weight. For bile duct ligation, the upper abdomen and peritoneum were then opened with a midline laparotomy and the common bile duct was exposed by caudal movement of the gut. The bile duct was then carefully separated from the flanking portal vein and the hepatic artery. The 6-0 suture was placed around the bile duct and secured with two surgical knots. A second cranial ligation was added in the same manner to prevent bile leakage, after which the abdomen was closed and animals were allowed to recover on a heat pad. For sham surgery, the bile duct was exposed but not ligated. When animals were ambulatory, PLGA (30 mg/kg, 0.3 mL) or normal saline (0.3 mL) were injected intravenously and then mice were euthanized 2 h later. Organs were extracted following extensive perfusion and imaged as described above.

### pH measurement of gastrointestinal compartments

PLGA degradation was first tested in vitro by measuring free lactate concentration after incubation 1 N sodium hydroxide for 30 min followed by neutralization with 1 N hydrochloric acid to pH 7.00 (controlled by a Thermo Scientific Orion 8103BNUWP ROSS Ultra Semi-Micro pH probe with a spherical tip). This allowed us to determine the degradation of approximately 30–40% of PLGA, as calculated from theoretical lactate content in intact PLGA nanoparticles (data not shown), measured using a commercial kit (Supplementary Table 5). Next, a single dose of these partially degraded PLGA nanoparticles was administered to C57BL/6 mice via oral gavage or injected IV, both at 30 mg/kg. Intact (non-degraded) PLGA nanoparticles given via oral gavage at the same dose served as a control (Supplementary Fig. 2). pH in GIT compartments from these experiment was assessed as described below.

In a separate set of experiments, C57BL/6 mice were fasted overnight (12 h) with free access to water. Following, they were either intravenously injected with 30 mg/kg PLGA NPs (n = 7) or saline (n = 5). After 12 h, all mice were euthanized by cervical dislocation, and their gastrointestinal tract was immediately removed and divided into six sections: stomach, duodenum, jejunum, ileum, caecum and colon. The contents of each compartment were flushed with 1 mL of deionized water, and the pH was determined using a pre-calibrated pH probe as described above (Supplementary Fig. 6).

### IPGTT and glycemia metrics

Mice were fasted (with access to water) for 12 h before an IP injection with glucose at 2 g/kg/body weight (no anesthesia given) at weeks 1 (prior to high-fat diet treatment), 5 (prior to PLGA nanoparticle injections) and 7 (after two weeks of PLGA injection and prior to euthanasia). Fasting blood glucose was obtained using a hand-held glucometer (Ascensia Diabetes Care, Parsippany, NJ) from tail-vein blood collected before gavage and re-sampled every 30 min for 2 h after the gavage. Fasting insulin was analyzed using a commercial ELISA kit (Supplementary Table 5) and HOMA-IR was calculated using the following formula: [HOMA-IR] = fasting blood glucose (mmol/L) × fasting insulin (mU/mL)/22.5^53^.

### Plasma determinations

Blood obtained from cardiac puncture after euthanasia was kept on ice and centrifuged for 25 min × 2,500 rpm at 4 °C in order to isolate plasma. Samples were stored at − 80 °C until further analysis. Lactate, insulin, cholesterol, TG and gram-negative bacterial endotoxin levels in plasma were determined using commercial kits (Supplementary Table 5). Serum levels of chemokine (C-X-C motif) ligand 1 (CXCL1/KC), CXCL5, interleukin (IL) 1ɑ, IL2, IL4, IL5, IL13, monocyte chemoattractant protein 1 (MCP-1) and macrophage inflammatory protein 2 (MIP-2) were determined using the Mouse High-Sensitivity Cytokine array (cat# MD31, Eve Technologies, Calgary, Canada).

### Glucose uptake, lactate release and insulin signaling experiments in differentiated L6 myotubes

L6 cells (ATCC CRL-1458) were first cultured in Dulbecco's Modified Eagle Medium (DMEM) supplemented with 10% fetal bovine serum, 1% penicillin–streptomycin (Pen/Strep) and 1% glutamine (2 mM) in a humidified 5% CO_2_ atmosphere at 37 °C. When cell density reached 80–90% confluence, cells were seeded in 12-well plates at 10^6^ cells/well (for insulin signaling/western blot) or in 96-well plates at 5 × 10^4^ cells/well (for 2-deoxy-D-( +)-glucose (2DG) uptake) in differentiation medium consisting of Minimum Essential Medium (MEM) with glutamine, 2% calf serum and 1% Pen/Strep, and the cells were cultured for 5–6 days with medium changes every other day. Next, the medium was replaced with fresh differentiation medium consisting of 1 mg/mL PLGA or the same volume of vehicle (PBS) and cultured for an additional 6 or 24 h following which the medium was replaced by MEM (without serum or supplements) and incubated for another 3–4 h.

#### Glucose uptake

At the end of the incubation period, 2DG uptake (96-well plates) was determined using a commercial kit according to manufacturer's instructions. Details of all reagents are provided in Supplementary Table 5.

#### Lactate release

L6 cells were cultured and treated with PLGA (1 mg/mL) in white opaque 96-well plates as described above followed by lactic acid analysis in cell lysates and supernatants using Promega Lactate-Glo bioluminescent assay and according to manufacturer's instructions (see Supplementary Table 5).

#### Western blot

Immunoblotting was performed according to previously described protocols and considerations^54^. Protein extracts were obtained through a cell lysis in sodium dodecyl sulfate (SDS) buffer consisting of 2.2% SDS, 100 mM dithiothreitol, 0.001% bromophenol blue, 5% Ficoll 400 (w/v), and 62.2 mM Tris at pH 6.8, plus a cocktail of protease and phosphatase inhibitors. Cell lysates were sonicated on ice (30 s, 100 W) and heat-denatured for 5 min at 95 °C. Following protein concentration analysis via IR spectroscopy (Direct Detect, Millipore-Sigma), protein extracts were loaded at 10–15 µg per lane on Bio-Rad Criterion TGX precast gels (10%) and subjected to electrophoresis in Tris–Glycine-SDS running buffer (25 mM Tris, 192 mM Glycine, 0.1% SDS, pH 8.3) at a constant voltage of 300 V. The separated proteins were transferred to polyvinylidene difluoride membranes using Bio-Rad Trans Blot Turbo apparatus and accompanying transfer reagents, according to the manufacturer’s instructions. The membranes were blocked for 1 h in 5% (w/v) non-fat dry milk in Tris-saline buffer with Tween 20 (TBS-T, 25 mM Tris–HCl, 154 mM NaCl, 0.05% Tween 20, pH 7.6). After washing in TBS-T (3 × 5 min), the membranes were probed overnight at 4 °C with primary antibodies diluted in TBS-T with 5% bovine serum albumin (BSA) as indicated in Supplementary Table 6A. The membranes were washed in TBS-T (3 × 5 min) followed by incubation with horseradish peroxidase (HRP)-conjugated secondary antibodies diluted in 5% BSA/TBS-T (Supplementary Table 6B) for 1 h at room temperature. Finally, the membranes were washed again (3 × 5 min) and exposed to enhanced chemiluminescence reagent consisting of 100 mM Tris–HCl pH 8.8, 1.25 mM luminol, 5.3 mM hydrogen peroxide and 2 mM p-iodophenylboronic acid as described previously^55^. The chemiluminescence signal was detected and recorded on Azure C400 multimodal imager (Azure Biosystems). The exposure time was experimentally determined so that the signal was not saturated using Azure cSeries software. Full blots can be found in Supplementary Fig. 7.

### Bacterial 16S rRNA sequencing

The sequencing and main bioinformatics analysis of bacterial 16S rRNA gene amplicons was carried out by Microbiome Insights (https://microbiomeinsights.com; Vancouver, BC, Canada) using whole cecums from mice excised at sacrifice. Briefly, DNA was extracted by placing samples into a MoBio PowerMag Soil DNA Isolation Bead Plate and bacterial 16S rRNA genes were amplified by PCR with primers targeting the V4 region. Amplicons were then sequenced using an Illumina MiSeq 250-bp paired-end kit (v.2) and sequences were clustered into 97%-similarity OTUs using the mothur software (https://www.mothur.org). An average of 26,463 quality-filtered reads were obtained per sample. Data analysis of data is further described below, and a more detailed description of methods is available upon request.

### Classification of microbial 16S sequences into operational taxonomic units

Classification of microbial 16S rRNA sequences into operational taxonomic units (OTU) was done using Qiime version 2019.1.0^56^. Sequences were demultiplexed and aligned to a 16S rRNA sequence database (Greengenes, version May 2013)^57^ and clustered into OTU at 97% sequence identity. We observed an average of 14,120 sequences per sample (see Supplementary Table 7 for the number of sequences per sample). A closed reference OTU picking strategy was followed. To account for biases caused by uneven sequencing depth, equal numbers of random sequences (10,224) were selected from each sample prior to calculating community-wide dissimilarity measures.

### Reconstruction of metagenomic pathways

The OTU table in a Biological Observation Matrix format was used for statistical analysis and illustration of results, and PICRUSt (phylogenetic investigation of communities by reconstruction of unobserved states) to predict the functional composition of metagenomes^58^. With PICRUSt, we were able to accurately map 16S microbial sequence reads to gene family abundances, by generating 95% confidence intervals for each gene prediction. These gene mappings were then used to reconstruct metagenomic pathways. The gene content estimations were mapped to KEGG pathways^59^ to identify the functional enrichment of these pathways, and the Wilcoxon Rank Sum test was used to determine significant differences. P values obtained where adjusted using the Bonferroni correction.

### Analysis of composition of microbiome

ANCOM is a statistical framework where the underlying structure in the microbiome data is used for comparing the composition of microbiomes in our treatment and control group^60^. ANCOM makes no distributional assumptions and can be implemented in a linear model framework. In this process, each sub-hypothesis is structured as follows: *H*_0(ij)_ : mean(log (x_i_/x_j_)) = mean(log (y_i_/y_j_)), where x_i_ denotes the i^th^ species abundances from samples x (i.e. treatment group), x_j_ denotes the j^th^ species abundances from samples x, and where y_i_ denotes the i^th^ species abundances from samples y (i.e. control group), y_j_ denotes the j^th^ species abundances from samples y. The W value reported as a result is the count of the number of times the null hypothesis *H*_0(ij)_ is rejected for the i^th^ species.

### Liver RNA isolation and transcriptome sequencing and analysis

Liver samples were grinded using the Geno/Grinder 2010 (SpexSamplePrep, Metuchen, New Jersey) for 90 s and 30 s rest at a rate of 1,500 strokes/min. RNA was isolated from the liver powder using the RNAeasy Mini Kit (Qiagen), quantified with the NanoDrop ND-1000 spectrophotometer (NanoDrop Technologies Inc., Wilmington, DE, USA) to ensure that the OD260/OD280 ratio was between 1.9 and 2.0, and checked for integrity with gel electrophoresis (1% agarose). RNA Integrity Number value was obtained for quality control with the Advanced Analytical Technologies Fragment Analyzer (Agilent Technologies, Inc., CA, USA) (RIN > 7.0), and RNAseq library preparation and sequencing were performed by Novogene using the Illumina HiSeq platform with the paired-end 150 bp sequencing strategy. For data analysis, Illumina bcl2fastq software was used to convert the raw bcl files into fastq files, then FastQC and trim_galore was used to examine the low-quality sequence fragments (Q25 cutoff). A total of 162 million reads were used, with an average of 27 million reads per sample. Next, an index sequence for STAR using the Gencode M13 reference feature that includes protein-coding genes as well as non-coding genes was built. In total, 50,600 genes were identified. Prior to sequence alignment, trim_galore (version 0.4.3) with the cutadapt package (version 1.12)^61^ was applied that removed any unnecessary genomic fragments (e.g. adapter dimers) and low quality nucleotide sequences from the raw reads. Raw sequencing reads to the mouse reference genome (mm10) were then mapped using STAR aligner^62^, and calculated the raw count using featureCounts package^63^. Gene level transcripts and Fragments Per Kilobase Million (FPKM) (N = 50,600) were extracted using RSEM package^64^. To test reproducibility and examine outlier samples, a principal component analysis (PCA) was conducted before proceeding with differentially expressed gene (DEG) analysis. A gene-by-sample matrix of reading counts was generated and analyzed using edgeR after removing unwanted variation (RUVg)^65^. The output of this analysis is a set of genomic regions that are significantly different between the experimental groups. Limma based edgeR method was used to determine differentially expressed genes, with a cutoff of log_2_ FC > 0.8, FDR < 0.05.

#### Enrichr links

The upregulated and downregulated gene sets were generated by extracting the 500 genes with the highest and lowest values, respectively, from the gene expression signature. These gene sets were subsequently submitted to Enrichr^66^, which is freely available at https://amp.pharm.mssm.edu/Enrichr/, using the gene set upload API.

#### Gene ontology enrichment analysis

Enrichment results were generated by analyzing the upregulated and downregulated gene sets using Enrichr. The following libraries were used for the analysis: GO_Biological_Process_2018, GO_Molecular_Function_2018, and GO_Cellular_Component_2018. Significant terms are determined by using a cut-off of *p* < 0.1 after applying Benjamini–Hochberg correction. For more information on the methods used to perform the enrichment analysis, see the Enrichr section.

#### Pathway enrichment analysis

Enrichment results were generated by analyzing the upregulated and downregulated gene sets using Enrichr. The following libraries were used for the analysis: KEGG_2016, Reactome_2016, and WikiPathways_2016. Significant terms are determined by using a cut-off of *p* < 0.1 after applying Benjamini–Hochberg correction. For more information on the methods used to perform the enrichment analysis, see the Enrichr section.

### Microarray data mining

Normal tissue gene expression data were obtained from Metabolic gEne RApid Visualizer (MERAV; https://merav.wi.mit.edu) ^30^, a web-based database that can query data from 4,300 microarrays. The expression data are normalized, which provides the opportunity to compare the expression between all samples in the database. Scripts for boxplots, heatmap and statistical analyses were implemented in R software.

### Statistical analysis

Differences between control and PLGA data is presented as a mean ± SEM. All samples were submitted to a Levene’s test for equality of variances and were analyzed using a two-tailed, independent *t*-test, unless stated otherwise (as per microbiome and RNAseq analysis). T-values and degrees of freedom are provided when comparisons were significant. Correlations were determined by Spearman’s rank correlation analysis. Statistical significance was assumed when *p* < 0.05 in all cases. IBM SPSS Statistics, version 25.0 (IBM Corp, USA) was used for statistical analyses and GraphPad Prism 7 was used to represent data.

## Supplementary information

Supplementary information.

Supplementary tables.

## Data Availability

RNA-seq raw data obtained from cecum microbiota (SUB5333382) and liver (SUB5359060) sequencing were deposited in the Sequence Read Archive of the NCBI (https://www.ncbi.nlm.nih.gov/sra). Other data that support the findings of this study are available from the corresponding author upon request.
