# Publisher Correction: Systemically-delivered biodegradable PLGA alters gut microbiota and induces transcriptomic reprogramming in the liver in an obesity mouse model

**DOI:** 10.1038/s41598-020-72446-0

**Published:** 2020-09-24

**Authors:** Alice Chaplin, Huiyun Gao, Courteney Asase, Palanivel Rengasamy, Bongsoo Park, Danielle Skander, Gürkan Bebek, Sanjay Rajagopalan, Andrei Maiseyeu

**Affiliations:** 1 School of Medicine, Cardiovascular Research Institute, Case Western Reserve University, 10900 Euclid Ave, Cleveland, OH 44106 USA; 2 Environmental Health and Engineering, Johns Hopkins Bloomberg School of Public Health, Johns Hopkins University, Baltimore, MD USA; 3 Department of Nutrition, Department of Electrical Engineering and Computer Science, Center for Proteomics and Bioinformatics, Case Western Reserve University, 10900 Euclid Ave, Cleveland, OH 44106 USA

Correction to: *Scientific Reports* 10.1038/s41598-020-69745-x, published online 14 August 2020

This Article contains errors in Figures 2, 3, 4 and 6 where the legends are incorrectly shown in black and grey. The correct Figures 2, 3, 4 and 6 with coloured legends are shown below as Figures 1, 2, 3, and 4 respectively.

**Figure 1:**
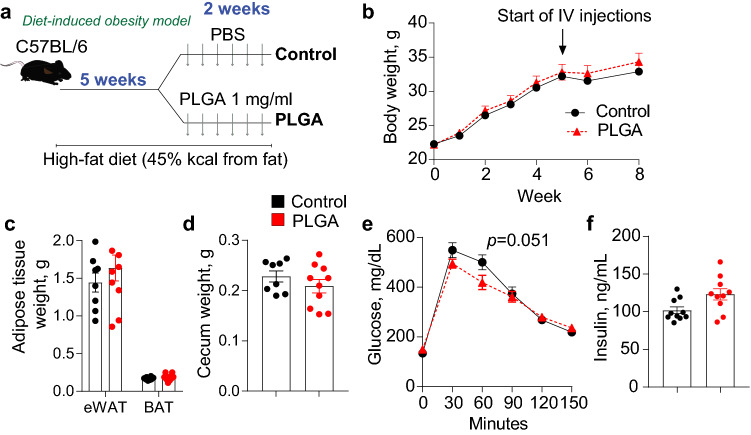
Effect of PLGA on body weight and glucose metabolism in diet-induced obese mice. (**a**) C57BL/6 mice were fed a high-fat diet for 5 weeks and were then injected IV with either PLGA nanoparticles or PBS six times during two weeks; (**b**) Body weight throughout experiment and (**c**) adipose tissue and (**d**) cecum weight at the end of the study were not significantly altered by treatment; (**e**) IPGTT before euthanasia revealed that PLGA nanoparticle-injected animals presented significantly better glucose clearance at 60 min (n = 10 mice/group); (**f**) Fasting insulin was not different between groups. n = 10 mice/group, independent *t*-test, *p* < 0.05.

**Figure 2:**
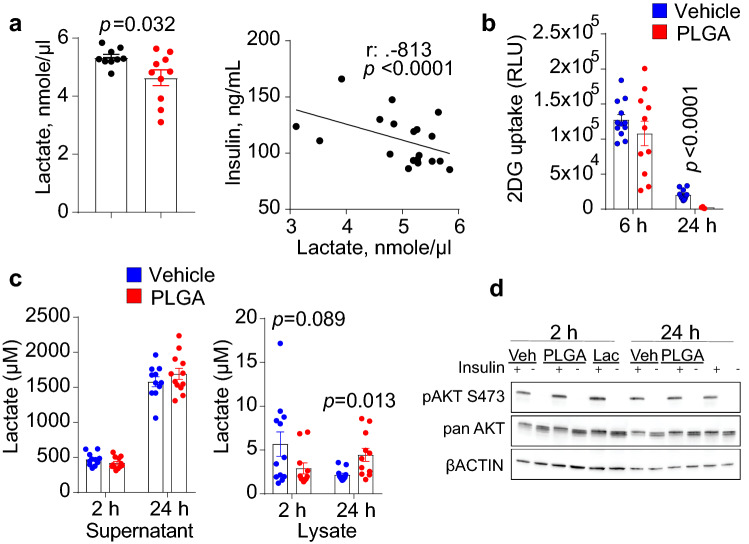
PLGA alters intracellular glucose uptake and lowers lactate levels. (**a**) Administration of PLGA nanoparticles for 2 weeks significantly lowered plasma lactate levels (*t-value*: 2.435; *degrees of freedom*: 11.528) (n = 10 mice/group). Furthermore, plasma lactate was negatively correlated with plasma insulin levels; (**b**) 2-deoxy-D-( +)-glucose (2DG) uptake in L6 myotube cells was significantly lower after 24 h treatment with PLGA (1 mg/mL) (*t-value*: 8.359; *degrees of freedom*: 13.698) (n = 12 replicates/group); (**c**) Lactate concentrations in supernatants and lysates of L6 myotubes were significantly higher in cell lysates after 24 h treatment with PLGA (1 mg/mL) (*t-value*: − 2.942; *degrees of freedom*: 11.476) (n = 12 replicates/group); (**d**) Insulin signaling experiments in L6 myotubes treated with 1 mg/mL PLGA, 50 mM lactate (Lac) or vehicle control (veh) showed no difference in levels of phosphorylated AKT in response to 100 nM insulin for 5 min. Representative images of phosphorylation on AKT residue Ser^473^ and loading controls, pan AKT and βACTIN are shown. Uncropped blot images are presented in supplementary files. Independent *t*-test was used when comparing two groups and correlation was determined by Spearman’s rank correlation analysis, *p* < 0.05.

**Figure 3:**
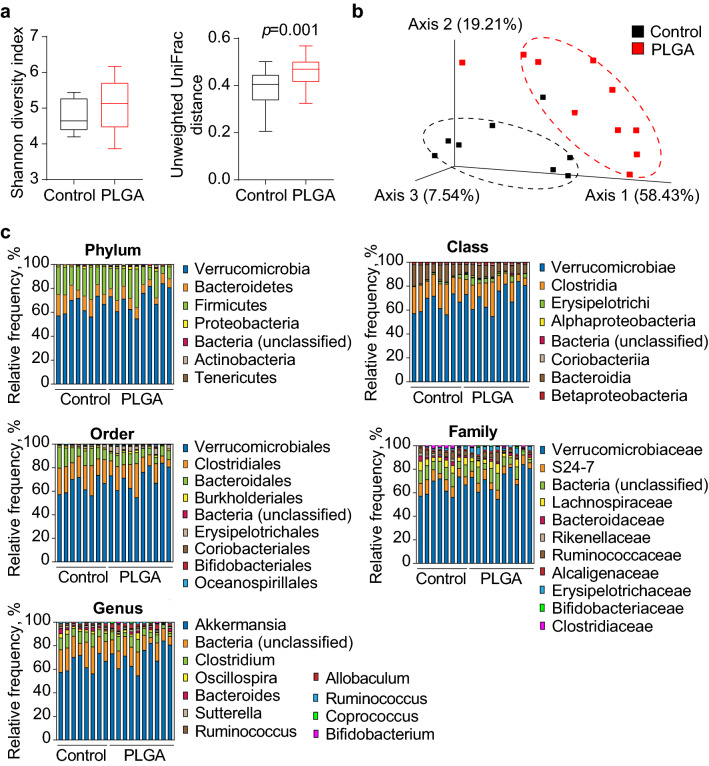
Bacterial diversity measurements show that gut microbiota diversity was affected by treatment. (**a**) Alpha diversity was determined using the Shannon diversity index on raw OTU abundance after filtering out contaminants (not significant, *p* = 0.286). However, when comparing phylogenetic tree information between groups using the unweighted unique fraction (UniFrac) distance measurement, there was a significant difference regarding gut microbiota diversity (pairwise Permanova) (n = 10/group); (**b**) Gut microbiota composition similarity among groups was represented using a principal coordinate ordination, based on weighted UniFrac distances, where points are individual samples; (**c**) Stacked column graphs show the relative frequency of bacterial species in control and PLGA mice in the gut microbiota of cecum feces, analyzed using Qiime2 Naive Bayes classifier using Greengenes (v13.5). Statistics: n = 10 mice/group, pairwise Kruskal–Wallis test (when comparing diversity indices), *p* < 0.05.

**Figure 4:**
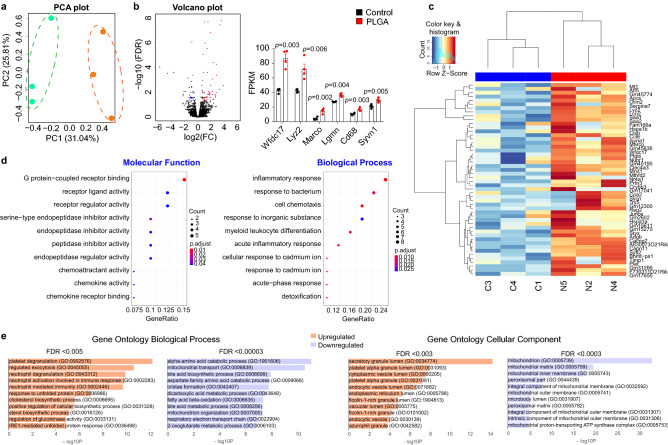
RNA-seq transcriptome analysis identifies metabolism, enzymatic degradation and cellular stress pathways in liver. (**a**) Principal component analysis (PCA) of whole-transcriptome RNA-seq read counts in liver. Dotted ellipses indicate the 95% confidence interval of samples that fall into two distinct groups (PLGA nanoparticle-treated and control). Axis percentages indicate variance in the data contribution (n = 3/group); (**b**) Volcano plot indicating the genes in liver with significantly increased (red dots) or decreased (blue dots) expression in PLGA treated group compared to control. The x-axis shows log2 fold-changes (FC) in the expression and the y-axis the log 10 false discovery rate (FDR) of a given gene being differentially expressed. Selected most significantly regulated genes are plotted in the bar graph as gene vs. fragments per kilobase of transcript per million mapped reads (FPKM): Wfdc17: activated macrophage/microglia WAP domain protein (*t-value*:  − 7.225; *degrees of confidence*: 3.414); Lyz2: lysozyme C-2 (*t-value*:  − 4.166; *degrees of confidence*: 6); Marco: macrophage receptor with collagenous structure (*t-value*: − 5.416; *degrees of confidence*: 6); Lgmn: legumain (*t-value*: − 7.369; *degrees of confidence*: 3.283); CD68: cluster of differentiation 68 (*t-value*: − 4.777; *degrees of confidence*: 6); Syvn1: Synoviolin 1 (*t-value*: − 4.286; *degrees of confidence*: 6); (n = 4 mice/group, independent *t*-test, *p* < 0.05); (**c**) Heatmap of hierarchical clustering indicates differentially expressed genes (columns) in liver from individual control (C3, C4, C1) and PLGA nanoparticle-treated animals (N5, N2, N4) samples (n = 3/group); (**d**) Gene ontology (GO) analysis presented as a scattergram of overrepresented GO terms in molecular function and biological process categories; (**e**) Additional GO analysis using more stringent FDR filtering (as indicated above the plot) demonstrated upregulation of various cell exocytosis and secretion pathways and downregulation of oxidative metabolism (mitochondrial function) pathways in liver.

