# Supplementary material for: Systemically-delivered biodegradable PLGA alters gut microbiota and induces transcriptomic reprogramming in the liver in an obesity mouse model
Source: Sci Rep. 2020 Aug 14;10:13786. doi: 10.1038/s41598-020-69745-x (PMC7429827; doi:10.1038/s41598-020-69745-x)
Supplement: Supplementary file 1 — Supplementary information. [file 41598_2020_69745_MOESM1_ESM.docx]

**Systemically-delivered biodegradable PLGA alters gut microbiota and induces transcriptomic reprogramming in the liver in an obesity mouse model**

**Authors:** Alice Chaplin^1^, Huiyun Gao^1^, Courteney Asase^1^, Palanivel Rengasamy^1^, Bongsoo Park^2^, Danielle Skander^3^, Gürkan Bebek^3^, Sanjay Rajagopalan^1^, Andrei Maiseyeu^1*^

**Affiliations:** ^1^Cardiovascular Research Institute, Case Western Reserve University, School of Medicine, 10900 Euclid Ave, Cleveland, OH 44106; ^2^Environmental Health and Engineering, Johns Hopkins Bloomberg School of Public Health, Johns Hopkins University, Baltimore, MD, USA; ^3^Center for Proteomics and Bioinformatics, Case Western Reserve University, Department of Nutrition, Department of Electrical Engineering and Computer Science, 10900 Euclid Ave, Cleveland, OH 44106.

**^*^ Corresponding author: Andrei Maiseyeu, axm1079@case.edu**

**Supplementary Figure 1**

**
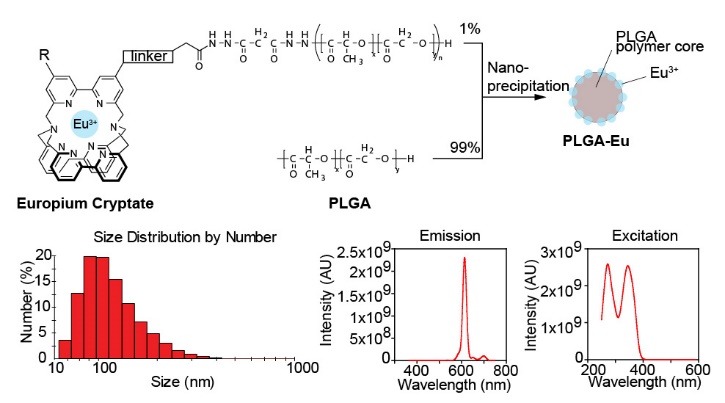
**

For imaging purposes, PLGA nanoparticles (NPs) were synthesized via nanoprecipitation from a polymer blend consisting of europium-cryptate-conjugated PLGA and carboxy-terminated PLGA as indicated (PLGA-Eu). PLGA nanoparticles had 92±14 nm hydrodynamic diameter (by dynamic light scattering, histogram) and exhibited typical Eu fluorescence profiles (excitation/emission plots).

**Supplementary Figure 2**

**
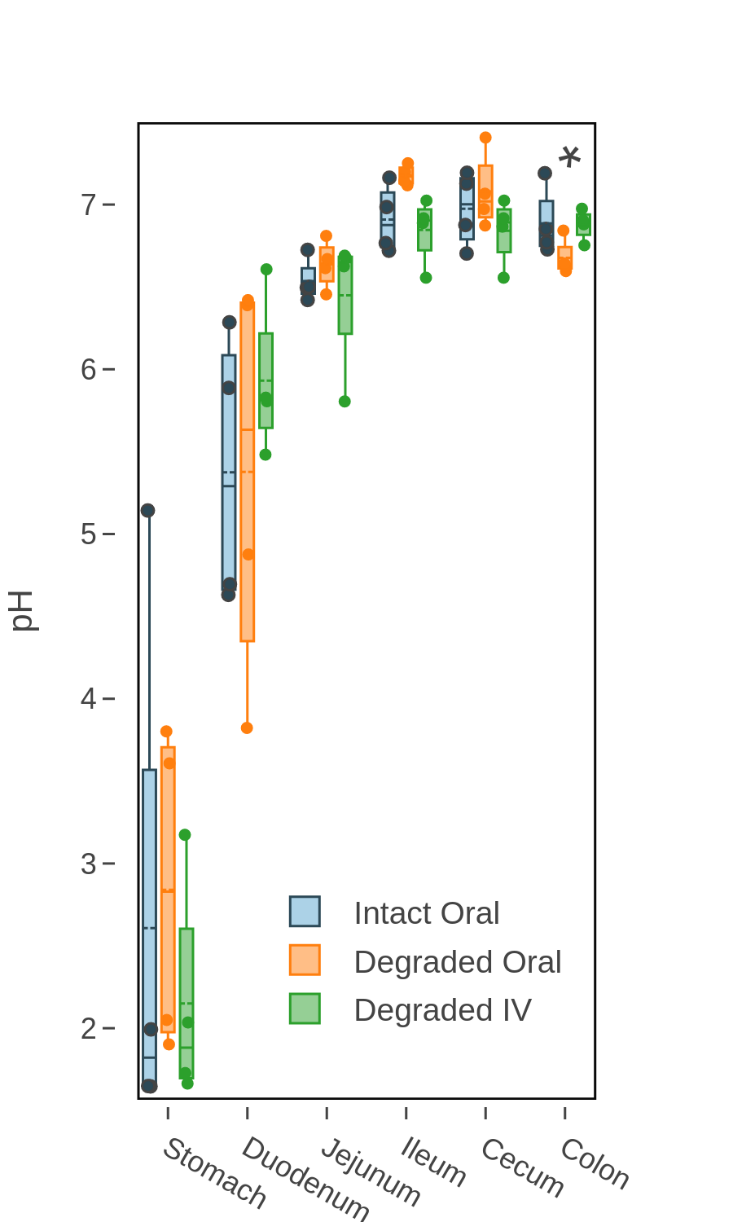
**

A single dose of either intact oral PLGA nanoparticles (in blue), degraded PLGA nanoparticles given orally (in orange) and intravenous degraded PLGA nanoparticles (in green) was administered to C57BL/6 mice. In colon, degraded oral PLGA nanoparticles decreased pH significantly compared to degraded IV PLGA NPs (*p*=0.032). Statistics: n=4/group, pairwise *t*-test, Bonferroni posthoc.

**Supplementary Figure 3**

**
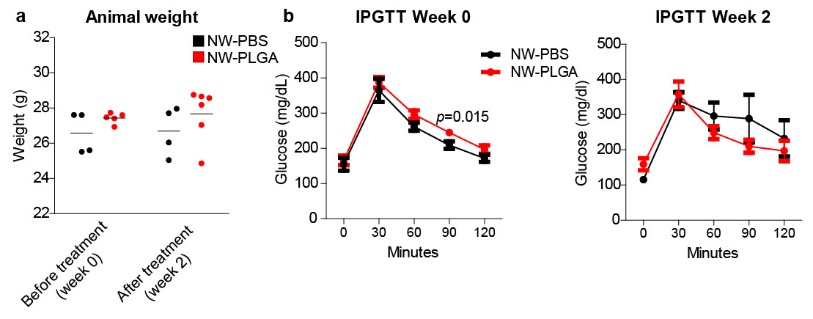
**

Normal weight, chow-fed C57BL/6 mice were injected with 30 mg/kg PLGA (n=6) (NW-PLGA) or saline (NW-PBS) (n=4) a total of six times in a two-week period (3 times/week). **a)** After two weeks, NW-PLGA showed no changes in body weight compared to NW-PBS animals; **b)** IPGTT was carried out in all animals prior to treatment (week 0) and at the end (week 2), showing no significant differences in glucose clearance by the end of treatment. Independent *t*-test was used when comparing two groups, *p*<0.05.

**Supplementary Figure 4**

**
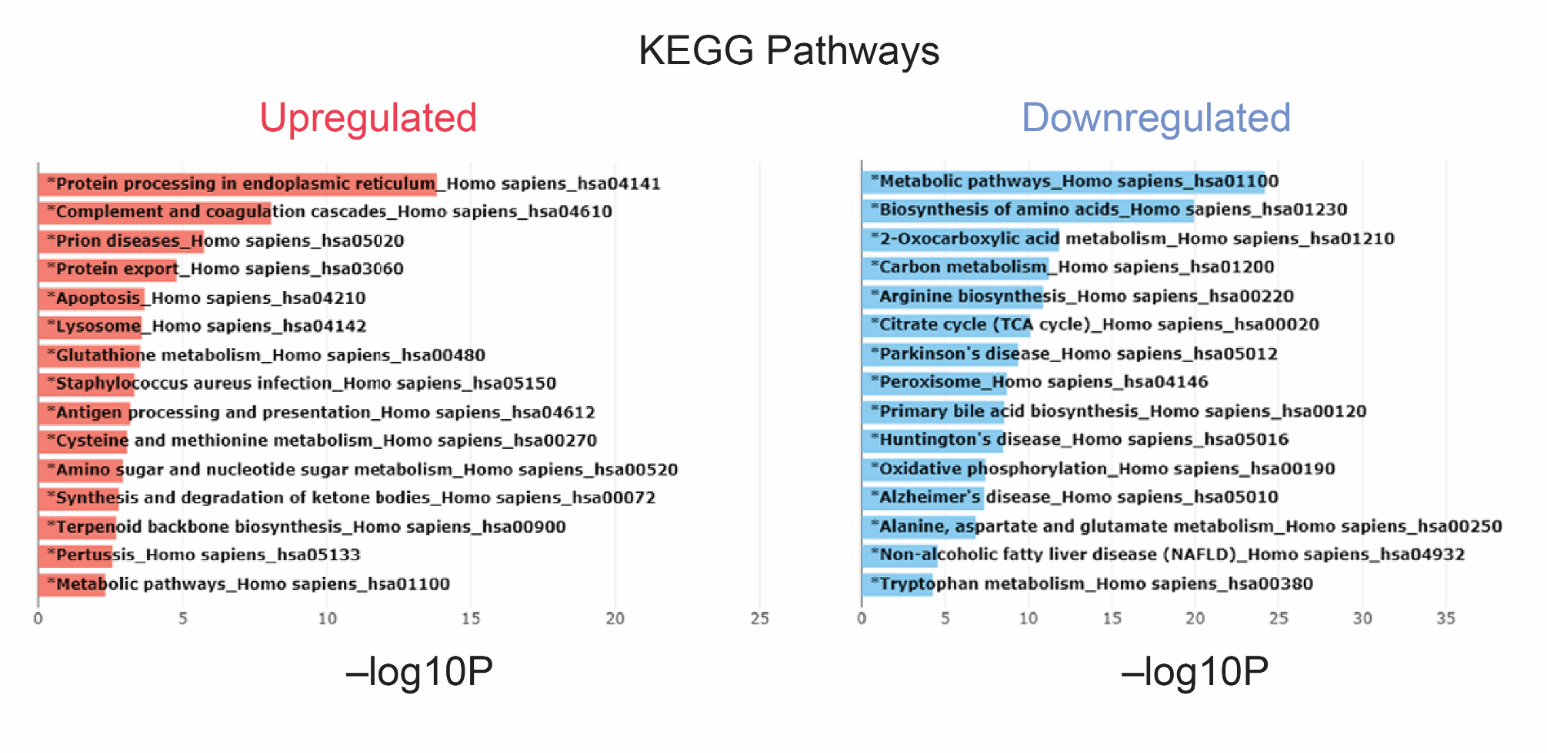
**

KEGG pathway analysis demonstrating significantly enriched pathways among the differentially expressed mRNAs in liver of PLGA vs. vehicle treated mice. KEGG: Kyoto Encyclopedia of Genes and Genomes database, <https://www.genome.jp/kegg/>.

**Supplementary Figure 5**


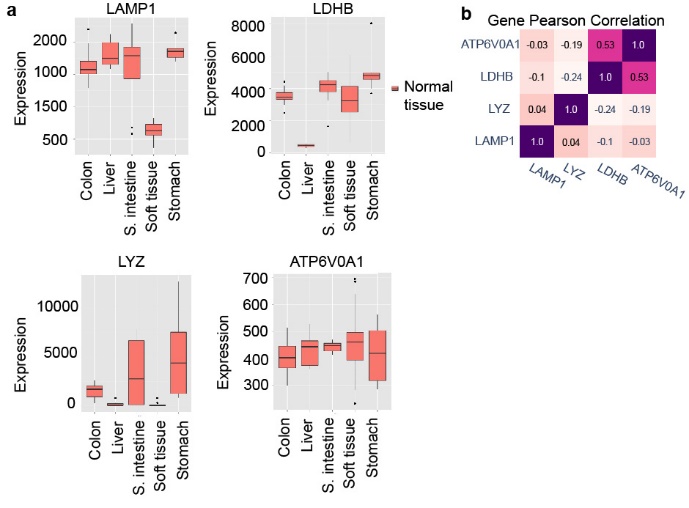


**a)** The expression of key lysosomal markers ATPase H+ transporting V0 subunit a1 (ATP6V0A1), lysosomal-associated membrane protein 1 (LAMP1) and lysozyme (LYZ) and their co-expression with lactate dehydrogenase B (LDHB) was determined from the Metabolic gEne RApid Visualizer MERAV database (http://merav.wi.mit.edu). Box plots represent the raw counts of expression levels (Y-axis) of the indicated genes in various tissues (X-axis) as indicated; **b)** Pearson’s correlation data derived from expression levels in normal tissues (analyzed as shown above in a) demonstrate strong correlation between LDHB and ATP6V0A1.

**Supplementary Figure 6**


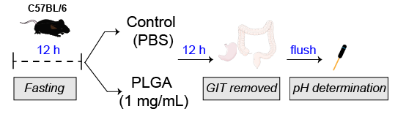


**Measurement of pH of GIT compartments.** C57BL/6 mice were fasted overnight (12 h) and intravenously injected with 30 mg/kg PLGA (n=7) or saline (n=5). After 12 h, all mice were euthanized and the pH of the different GIT compartments was determined (as detailed in the Methods section)**.**

**Supplementary Figure 7**

**
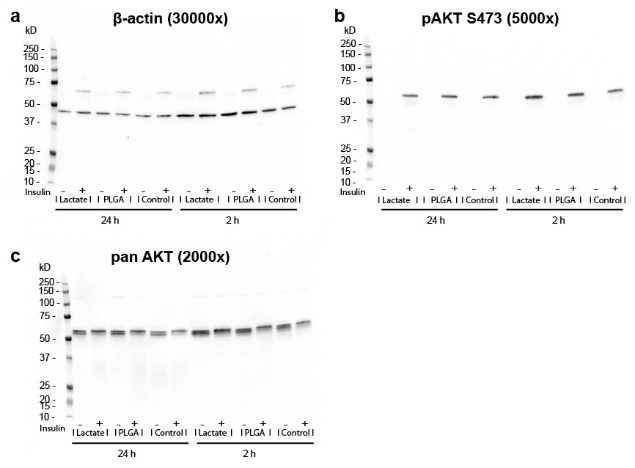
**

Uncropped Western Blot images from Figure 3D of the main manuscript.

**Supplementary Table 1**

Analysis of composition of microbiomes results.

| **Group** | **Control** | | | | | **PLGA** | | | | |
| --- | --- | --- | --- | --- | --- | --- | --- | --- | --- | --- |
| **Percentile** | **0** | **25** | **50** | **75** | **100** | **0** | **25** | **50** | **75** | **100** |
| **Bacteroidetes** | 976 | 1494 | 1717 | 2094 | 3076 | 718 | 967.25 | 1067.5 | 1096.5 | 1737 |
| **Firmicutes** | 2120 | 2655.75 | 2993 | 3449.5 | 3981 | 1242 | 1561.5 | 2343 | 2816 | 3726 |

**Supplementary Table 4**

PLGA nanoparticles used.

| **PLGA polymer** | **Source** | **Identifier or catalog number** |
| --- | --- | --- |
| mPEG-b-PLGA (PEG M_n_ 2000, PLGA M_n_ 4500) | Sigma-Aldrich | 764825 |
| Acid terminated PLGA (M_w_ 7,000-17,000), Resomer RG 502 H | Sigma-Aldrich | 719897 |
| PLGA-NH2 (50:50 LA:GA, 28,000 Da) | PolySciTech | Al10 |

**Supplementary Table 5**

Reagents and materials used.

| **Reagent or material** | **Source** | **Identifier or catalog number** |
| --- | --- | --- |
| Mouse Insulin ELISA | ALPCO | 80-INSMS-E01 |
| Pierce™ LAL Chromogenic Endotoxin Quantitation Kit | ThermoFisher Scientific | 88282 |
| Lactate Assay Kit | Millipore-Sigma | MAK064-1KT |
| Cholesterol Standard (200 mg/dL) | Stanbio Laboratory | 1012-030 |
| Infinity TM Cholesterol Liquid Stable Reagent kit | Fisher Scientific | TR13421 |
| Triglycerides Standard 2 mg/mL | Pointe Scientific | T7531-STD |
| Pointe Scientific Triglycerides (GPO) (Liquid) Reagent Set | Fisher Scientific | T7532500 |
| DMEM | Corning | 10-017-CM |
| MEM | Thermo Fisher | 11095072 |
| Calf serum | Colorado Serum Company | 31334 |
| Glucose-Glo Assay | Promega | J1342 |
| Lactate-Glo Assay | Promega | J5021 |

**Supplementary Table 6**

List of antibodies used for Western Blot.

**a)**

| **Antigen** | **Primary antibodies** | | **Dilution** | |
| --- | --- | --- | --- | --- |
|  | **mAb clone** | **Manufacturer/Cat#** | **Primary antibodies** | **Secondary antibodies** |
| beta-ACTIN | BA3R (mouse) | Thermo /MA515739 | 1:10,000 | 1:100,000 |
| PhosphoAKT Ser473 | D9E (Rabbit) | Cell Signaling/4060S | 1:5000 | 1:50,000 |
| Akt (pan) | 40D4 (Mouse) | Cell Signaling/4298S | 1:2000 | 1:50,000 |

**b)**

| **Secondary antibodies** | |
| --- | --- |
| **Name - conjugate** | **Manufacturer/Cat#** |
| Donkey anti-Mouse-HRP | ThermoFisher Scientific/A16017 |
| Donkey anti-Rabbit-HRP | ThermoFisher Scientific/A16035 |

**Supplementary Table 7**

Sequence count for cecum microbiota analysis.

| **Sample ID** | **Sample class** | **Counts** |
| --- | --- | --- |
| C1 | Control | 17,195 |
| C2 | Control | 13,166 |
| C3 | Control | 13,749 |
| C4 | Control | 16,866 |
| C5 | Control | 12,841 |
| C6 | Control | 14,446 |
| C7 | Control | 17,368 |
| C8 | Control | 11,573 |
| P1 | PLGA | 14,451 |
| P2 | PLGA | 10,224 |
| P3 | PLGA | 13,491 |
| P4 | PLGA | 11,758 |
| P5 | PLGA | 11,483 |
| P6 | PLGA | 14,791 |
| P7 | PLGA | 12,787 |
| P8 | PLGA | 13,591 |
| P9 | PLGA | 20,244 |
| P10 | PLGA | 14,142 |
